# Solanaceae Family Phytochemicals as Inhibitors of 3C-Like Protease of SARS-CoV-2: An In Silico Analysis

**DOI:** 10.3390/molecules27154739

**Published:** 2022-07-25

**Authors:** Raisul Awal Mahmood, Anamul Hasan, Mohammed Rahmatullah, Alok K. Paul, Rownak Jahan, Khoshnur Jannat, Tohmina Afroze Bondhon, Tooba Mahboob, Veeranoot Nissapatorn, Maria de Lourdes Pereira, Tridib K. Paul, Ommay Hany Rumi, Christophe Wiart, Polrat Wilairatana

**Affiliations:** 1 Department of Chemistry, Bangladesh University of Engineering & Technology, Dhaka 1000, Bangladesh; raisulawal@gmail.com; 2 Department of Biotechnology & Genetic Engineering, University of Development Alternative, Dhaka 1207, Bangladesh; anamulhasanoris@gmail.com (A.H.); alok.paul@utas.edu.au (A.K.P.); rownak86@hotmail.com (R.J.); jannat.koli.22@gmail.com (K.J.); afrozebondhon@gmail.com (T.A.B.); paul.kumarov@gmail.com (T.K.P.); ommay.hany@bge.uoda.edu.bd (O.H.R.); 3 School of Pharmacy and Pharmacology, University of Tasmania, Hobart, TAS 7001, Australia; 4 School of Allied Health Sciences and World Union for Herbal Drug Discovery (WUHeDD), Walailak University, Nakhon Si Thammarat 80160, Thailand; tooba666@hotmail.com (T.M.); nissapat@gmail.com (V.N.); 5 Department of Medical Sciences, CICECO-Aveiro Institute of Materials, University of Aveiro, 3810-193 Aveiro, Portugal; 6 Institute for Tropical Biology and Conservation, Universiti Malaysia, Kota Kinabalu 88400, Sabah, Malaysia; asianjpharmcog@gmail.com; 7 Department of Clinical Tropical Medicine, Faculty of Tropical Medicine, Mahidol University, Bangkok 10400, Thailand; polrat.wil@mahidol.ac.th

**Keywords:** COVID-19, SARS-CoV-2, Solanaceae, incanumine, anthocyanins

## Abstract

COVID-19, caused by the coronavirus SARS-CoV-2, emerged in late December 2019 in Wuhan, China. As of 8 April 2022, the virus has caused a global pandemic, resulting in 494,587,638 infections leading to 6,170,283 deaths around the world. Although several vaccines have received emergency authorization from USA and UK drug authorities and two more in Russia and China, it is too early to comment on the prolonged effectiveness of the vaccines, their availability, and affordability for the developing countries of the world, and the daunting task to vaccinate 7 billion people of the world with two doses of the vaccine with additional booster doses. As a result, it is still worthwhile to search for drugs and several promising leads have been found, mainly through in silico studies. In this study, we have examined the binding energies of several alkaloids and anthocyanin derivatives from the Solanaceae family, a family which contains common consumable vegetables and fruit items such as eggplant, pepper, and tomatoes. Our study demonstrates that Solanaceae family alkaloids such as incanumine and solaradixine, as well as anthocyanins and anthocyanidins, have very high predicted binding energies for the 3C-like protease of SARS-CoV-2 (also known as Mpro). Since Mpro is vital for SARS-CoV-2 replication, the compounds merit potential for further antiviral research towards the objective of obtaining affordable drugs.

## 1. Introduction

Coronaviruses are so named because of the spike-like proteins on their surface resembling a corona. Thus far, seven coronaviruses have been identified, which can affect humans. Two of them, identified in the 1960s, are HCoV-OC43 and HCoV-229E, causing common colds [1,2,3]. Two other coronaviruses affecting humans are HCoV-NL63 and HCoV-HKU1; they can cause fever, cough, and rhinorrhea [4]. Overall, it can be said that these four coronaviruses mostly cause only mild symptoms in human beings, which go away within a few days. However, this premise does not hold for three other coronaviruses, which have emerged in this century. The severe acute respiratory syndrome (SARS) virus first emerged in 2002 in China; the Middle East respiratory syndrome (MERS) virus emerged in the Middle Eastern countries in 2012, while the severe acute respiratory disease virus 2 (also known as SARS-CoV-2, causing the disease known as COVID-19) emerged in late December 2019 in Wuhan, China [5].

It is important to realize that all three viruses had and still have the potential to cause pandemics; however, luckily for human beings, the first two disappeared as suddenly as they emerged causing some, but not severe numbers of fatalities and minimal disruptions in the economy of people and the world as a whole. That has not been the case with COVID-19. As of 8 April 2022, the virus has caused a global pandemic, resulting in 494,587,638 infections leading to 6,170,283 deaths around the world. The world economy has been more or less shattered because of the frequent lockdowns, quarantines, maintaining distance, and simply the closure of small businesses as well as the lay-offs that occurred in practically every type of production and other units (including tourism, aviation, and so forth). There is a consensus among economists that the current pandemic will plunge the world into a global recession [6].

Very recently, four vaccines for SARS-CoV-2 were prepared by Pfizer-BioNTech (USA-Germany joint venture, New York, NY, USA), Moderna (Cambridge, MA, USA), Johnson & Johnson (New Brunswick, NJ, USA), and AstraZeneca-University of Oxford (UK–Sweden joint venture, Cambridge, UK) and have been approved or are on the verge of approval on an emergency basis for administration by the Food and Drug Administration of the USA and respective authorities in the USA and UK. Russia and China have prepared their own vaccines with not much information disclosed so far, except that they are “safe”. Despite the enormous potential of the vaccines already developed and more vaccines in the process of development, questions have already arisen as to the long-term effects of the vaccines, availability of vaccine storage refrigeration units in the less developed countries (Pfizer-BioNTech and Moderna vaccines would need at least −70°C and −20°C, respectively for storage), and an answer to the question as to who is going to pay for the vaccine costs. To these questions must be added the daunting responsibility of twice dosing 7 billion people of various races and creeds in both accessible and remote parts of the world and with differing sorts of beliefs as to whether injecting themselves with vaccines is permissible or not. To this, it has to be added that now it has been further found that booster doses of all vaccines need to be administered because of the emergence of newer variants of SARS-CoV-2; it seems that the world population now has to be vaccinated at least three times—two doses followed by a booster dose.

Anti-COVID-19 drugs can provide an easy solution to the problem, provided that suitable drugs can be discovered in the first place. Other than doing costly experiments for antiviral activity in only a limited number of appropriate biosafety laboratories with potentially millions of compounds, scientists have taken the more pragmatic approach of performing in silico interactions with various targets present in SARS-CoV-2. One of the most important targets thus far identified in SARS-CoV-2 is the 3C-like protease, a chymotrypsin-like protease also known as Mpro or the main protease, which plays a vital role in viral replication [7]. A number of promising results have come out, suggesting as to which directions future research may take place. Herbal medicines and phytochemicals are considered good candidates for inhibition of SARS-CoV-2 and China and South Korea have already issued traditional medicine treatment guidelines [8].

The World Health Organization (WHO) in their latest guideline (published 3 March 2022) on therapeutics for COVID-19 has conditionally recommended the antiviral drug molnupiravir, recommended the Janus kinase inhibitor drugs—baricitinib, ruxolitinib, and tofacitinib, conditionally recommended the monoclonal antibody drugs sotrovimab and casirivimab-imdevimab, recommended the interleukin-6 (IL-6) receptor blockers tocilizumab or sarilumab, and conditionally did not recommend the use of the antiviral drug remdesivir and the antiparasitic drug ivermectin [9]. However, as described in the WHO guideline brochure [9], even these recommended drugs cannot be used for all COVID-19 patients and all of them have adverse side effects. Additionally, these drugs do not come cheaply. For instance, in Bangladesh a market survey by the authors in the capital city Dhaka showed that a 10-pack of baricitinib costs Bangladesh taka (BDT) 200 (USD 1 = BDT 85), while molnupiravir costs BDT 2800 for a full course. A typical day laborer in Dhaka City earns USD 3–4 daily.

As a consequence, scientists have turned their attention to plants and traditional medicinal systems such as Traditional Chinese Medicine (TCM) for an affordable and safe prophylactic/therapeutic against COVID-19, a viewpoint further endorsed by WHO [10]. As early as 2020, a review article pointed out the use of herbal medicines in various parts of the world, caused possibly due to the lack of any effective drugs against this new disease [11]. Since then, a large number of papers have come out demonstrating the in silico potential of plants/plant extracts and various phytochemical types on inhibition of COVID-19 which also provided additional scientific values to this present study.

The plants *Withania somnifera* (L.) Dunal, *Tanacetum parthenium* L., and *Ammoides verticillata* (Desf.) Briq, and *Nigella sativa* L. have been reported in a review as being effective for the treatment and/or prevention of COVID-19. The same review [12] mentions the possible use of several phytochemicals in the treatment of COVID-19 such as “aloin and terpenes as antiseptics; isothymol, dithymoquinone, and glycyrrhizin as inhibitors of virus binding and entry; glycyrrhizin and berberine as replication suppressants; ginsenoside Rg1 and parthenolide as immunomodulators; eriocitrin, rhoifolin, hesperidin, naringin, rutin, and veronicastroside as anti-complements”. Another review has mentioned the potential of various alkaloids (in silico studies) as anti-COVID-19 agents. Some of these alkaloids are berbamine, cepharanthine, conessine, fangchinoline, harmine, lycorine, and tylophorine. The in silico activity studies have shown their possible uses for blocking the E proteins, blocking the expression of S and N proteins as well as RdRp inhibitor, Mpro inhibitor, blocking the expression of S and N proteins, Mpro inhibitor, Mpro inhibitor, and Mpro inhibitor plus blocking the S and N proteins, respectively [13].

Flavonoids appear to be a group of phytochemicals having promise as therapeutics/prophylactics against COVID-19 [14,15,16]. In fact, flavonoids have been mentioned as “a complementary approach to conventional therapy of COVID-19”. A large number of flavonoids and their subgroups (such as flavanes, flavanoles, flavonoles, and flavanones, as well as other subgroups) have been listed by Solnier and Fladerer [17]. The list includes compounds such as hesperidin, apigenin, luteolin, rhoifolin, kaempferol, myricetin, quercetin, and diadzein, among others. Another review listed more than fifty flavonoid group of compounds; most studies have been done in silico with Mpro (otherwise known as 3CLpro) of SARS-CoV-2 [18]. Several dozen flavonoid group compounds have been listed by Tabari and colleagues; in silico studies with these flavonoids have been done with various components of SARS-CoV-2 and related viruses [19].

Towards finding an affordable drug candidate for COVID-19, we had been screening phytochemicals in silico with the 3C-like protease of SARS-CoV-2 as our target [20,21,22,23,24,25,26,27,28]. Our studies indicated that certain phytochemicals from *Solanum surattense* Burm.f. (Solanaceae) have the lowest predicted binding energies for Mpro of SARS-CoV-2 in molecular docking studies conducted in silico [29]. In particular, the lowest predicted binding energy of −10.8 kcal/mol was obtained with an alkaloid, alpha-solamargine. Solanaceae family plants yield several widely consumed fruits and vegetables such as eggplants, peppers, and tomatoes (from *Solanum melongena* L., *Capsicum annuum* L., and *Solanum lycopersicum* L., respectively). It was of interest to screen a number of alkaloids, and other bioactive compounds such as anthocyanins and anthocyanidins found in Solanaceae family plants for their in silico binding affinities to Mpro of SARS-CoV-2 (it should be noted that binding energies of all compounds were determined initially in molecular docking studies with structures obtained from PubChem; those of anthocyanins/anthocyanidins were further evaluated taking into account the effect of pH and the various structural forms of these compounds, such as hemiacetal, hemiketal, chalcone, and quinoidal forms).

The hemiacetal (hemiketal) form of the anthocyanins is known to predominate in pH 4–5 which can undergo ring opening to form cognate E and Z chalcones. In a pH physiologically relevant for humans (pH 7.4), the predominant species of anthocyanins are reported to be the quinoidal anions.

Anthocyanins exist in an aqueous phase in a mixture of four molecular species, the concentrations of which depend on the pH [30]. At pH 1–3 the flavylium cation is red, at pH 4–5 the carbinol pseudobase (pb) generated is colorless, and at pH 7–8 the quinoidal-base (qb) formed is blue-purple, which could turn into a chalcone.

In grapes and wines, the anthocyanins are in flavylium form. However, during digestion, they may reach higher pH values, forming the carbinol pseudobase, quinoidal-base, or chalcone, and these compounds appear to be the potential forms to be absorbed from the gut into the blood system [31]. Chalcone gives trans-chalcone (Ct) by isomerization, a process that could occur in a few seconds or hours and even days and depends on the presence and positions of various functional groups in the rings. For that reason, molecular docking studies were also carried out with hemiacetal, hemiketal, chalcone, and quinoidal forms of anthocyanins/anthocyanidins to get an overall view of not only any molecular docking and binding energy differences, but also to see how pH affects molecular docking interaction with Mpro amino acids with various pH-dependent forms of the compounds.

## 2. Methods

### 2.1. Molecular Docking

A number of crystal structures of SARS-CoV-2 M^pro^ in complex with various compounds or potential inhibitors can be obtained from the Protein Data Bank: (A) 13b (PDB entry: 6Y2G, 2.20 Å resolution); (B) Michael acceptor N3 (PDB entry: 6LU7, 2.16 Å resolution); (C) Carmofur (PDB entry: 7BUY, 1.60 Å resolution); (D) 11a (PDB entry: 6LZE, 1.505 Å resolution); (E) 11b (PDB entry: 6M0K, 1.504 Å resolution); (F) GC373 (PDB entry: 6WTK, 2.00 Å resolution); (G) GC376 (PDB entry: 6WTT, 2.15 Å resolution); (H) Q5T (PDB entry: 6Z2E, 1.70 Å resolution); (I) X77 (PDB entry: 6W63, 2.10 Å resolution).

We have taken Mpro (Pdb: 6LU7) as our target protein, which has an inhibitor known as N3 and has been published before [32]. Mpro (6LU7) is one of the most used crystal structures in molecular docking studies; a PubMed Central search with the term <Mpro molecular docking> produced 1021 hits. For most docking preparation, we removed the water molecule from the crystallographic structure of Mpro and also removed the N3 molecule, but in some studies, we compared the results with molecular docking studies of Mpro with attached N3. We added the polar hydrogen atom because crystallographic structures usually lack a hydrogen atom. The addition of a polar hydrogen atom and removal of water molecules and N3 were done with Pymol software. We have used here a blind docking method using the program AutoDock Vina for screening phytochemicals, which can be good inhibitors. So, the grid box in AutoDock Vina was generated aiming to cover up all the key residues for ligand binding of the main protease, where the center was at X: −20.82, Y: 12.49, and Z: 38.77 and the dimensions of the grid box were, X: 67.69, Y: 81.35, and Z: 107.01 (unit of the dimensions, Å). We have used exhaustiveness “16” for better ligand and protein binding. The predicted binding affinity values are an average of values from five independent runs of the docking program. AutoDock Vina provides a total of nine docked poses for each ligand, and among them pose 1 is the best pose with highest binding affinity. We have saved pose 1 in pdb format by using Pymol for further analysis. The 2D diagram and interactions between ligand and amino acids of protein were depicted from Discovery Studio Software. Furthermore, to validate our molecular docking results, we docked the original removed inhibitor N3 back to Mpro (6LU7) to determine whether N3 bound to Mpro in a similar manner as before.

For comparative studies with some selected compounds, we have also used another crystalline structure of the Mpro complexed with an inhibitor 11a (PDB entry: 6LZE, 1.505 Å resolution) [33]. The inhibitor was removed prior to molecular docking studies. The addition of polar hydrogen atoms and removal of water molecules and inhibitor 11a were done with Pymol software. The grid box in AutoDock Vina was generated aiming to cover up all the key residues for ligand binding of the main protease, where the center was at X: −18.29, Y: 13.85, and Z: 40.58 and the dimensions of the grid box were, X: 101.33, Y: 96.21, and Z: 126.25 (unit of the dimensions, Å). Other procedures were as described before for 6LU7 [34].

Twenty phytochemicals present in Solanaceae family plants were studied for their binding affinity to Mpro. As shown in Table 1, all the phytochemicals were obtained from the Solanum genus of the Solanaceae family plant group. Several control compounds (used as antiviral/repurposed drugs and phytochemicals) were also subjected to molecular docking studies to compare binding energies. Ligand molecules downloaded from PubChem [34] in sdf format were optimized with the force field type MMFF94 using Openable software and saved as pdbqt format. Ligand binding to Mpro was carried out through blind molecular docking using AutoDock Vina [35]. The predicted binding affinity values are an average of values from five independent runs of the docking program. Diagrams and interactions between ligand and amino acids of protein were depicted from Discovery Studio Software [36].

### 2.2. Lipinski’s Rule of Five

Lipinski’s rule of five or Ro5 [37] was followed to predict the drug-like properties of the phytochemicals of the Solanaceae family. The rule mentions that poorly absorbed molecules by the intestinal wall would present two or more of these characteristics: molecular weight over 500, lipophilicity (log P ˃ 5), hydrogen bond (HB) donor groups (expressed as the sum of OHs and NHs groups) more than 5, more than 10 HB acceptor groups (expressed as the sum of Os and Ns atoms), and molar refractivity outside a range of 40–130 [38].

### 2.3. Molecular Dynamics

A molecular dynamics (MD) simulation was employed to validate the docking results to execute the best phytochemical drug. A 100 ns MD simulation was performed for the main protease in apo form (without drug) and holo-form (drug–protein). AMBER14 force field was applied over the course of simulation [37]. The total system was equilibrated with 0.9% NaCl at 298 °K temperature in the presence of water solvents at pH 7.4. During the simulation, a cubic cell was generated within 8 A° on each side of the system where periodic boundary condition was considered. A time step of 1.25 fs was maintained to proceed the whole simulation and the snapshots were taken at every 100 ps. The whole MD simulation of 100 ns was executed using YASARA Dynamics program [38]. Several data including root mean square deviation (RMSD) values for alpha carbon, backbone, and heavy atoms, radius of gyration (Rg), solvent-accessible surface area (SASA), and molecular surface area (MolSA) were obtained from MD simulations using macro files.

## 3. Results and Discussion

**Figure 1:**
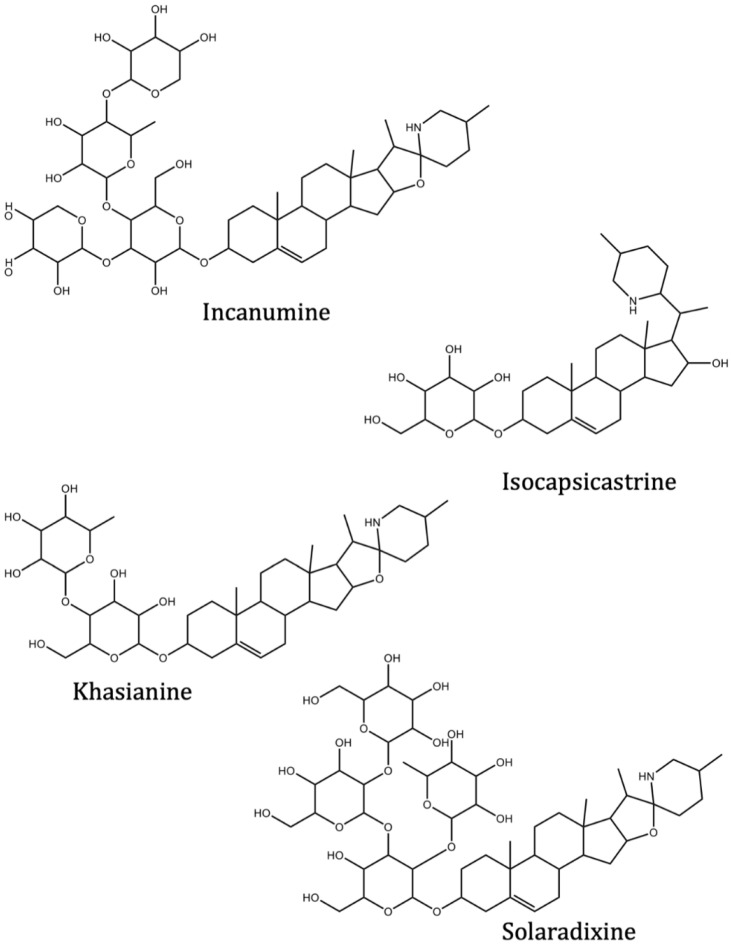

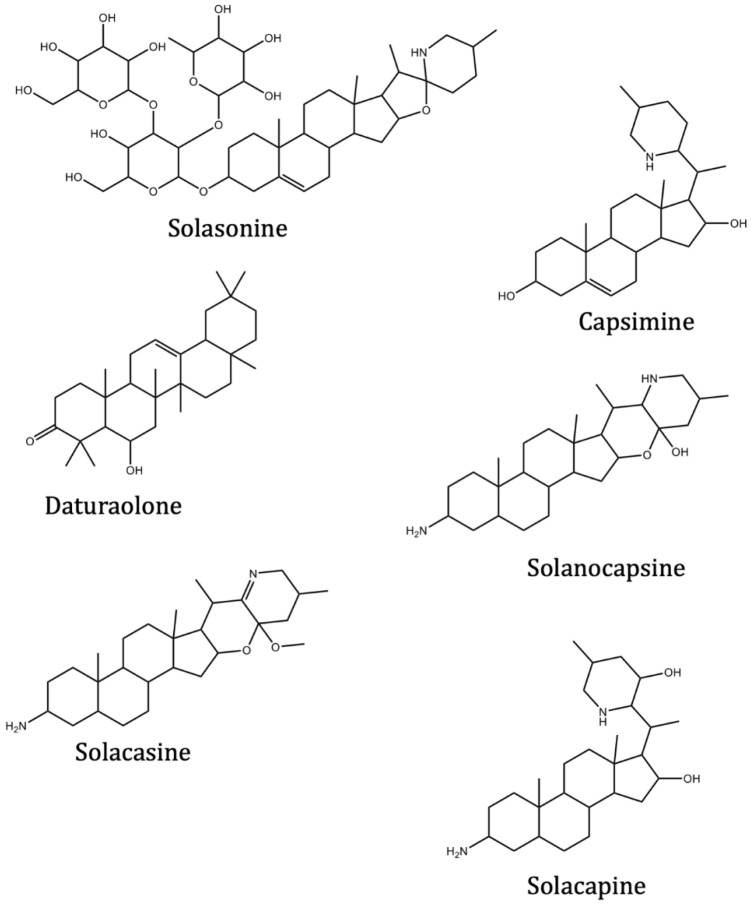

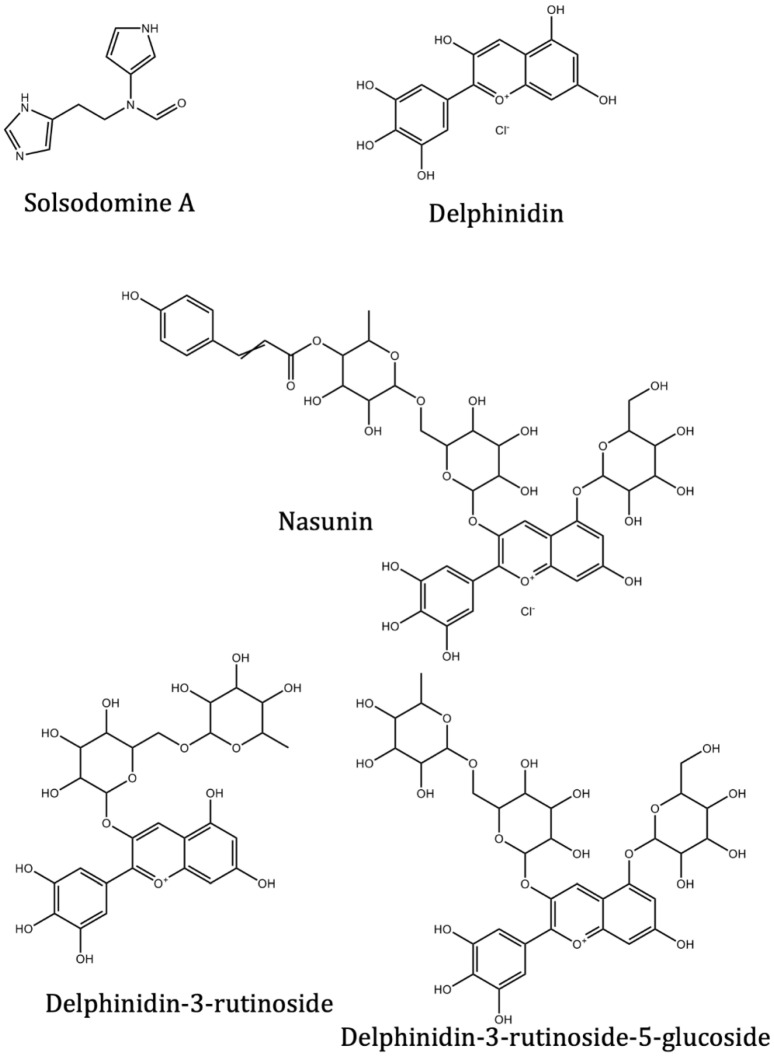

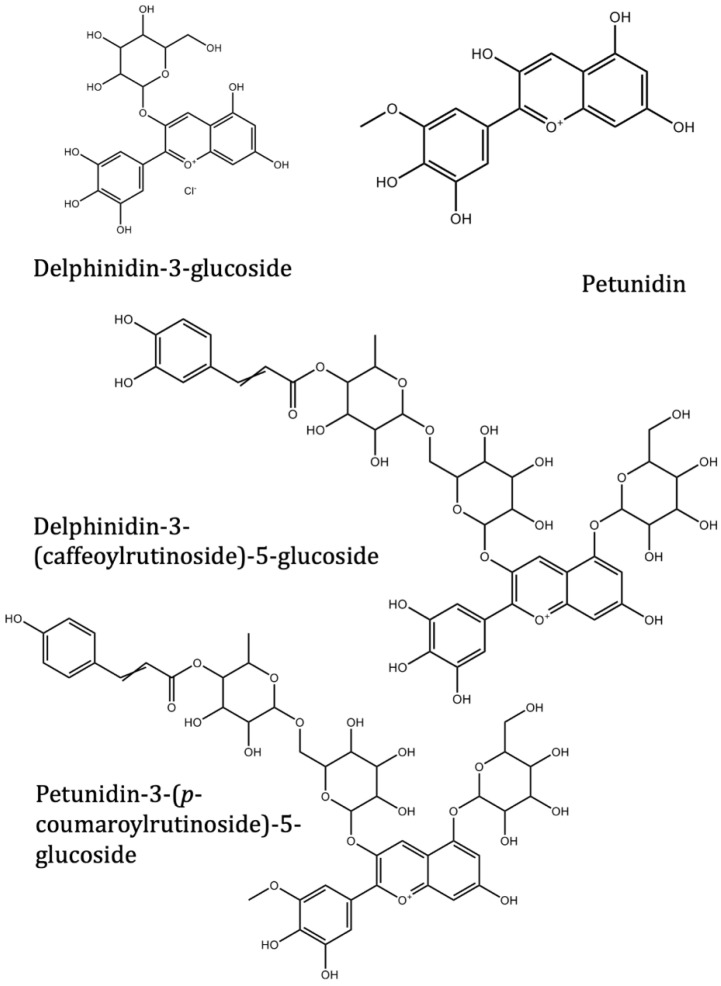
Structure of the Solanaceae family phytochemicals in the present study.

The predicted binding energies of the twenty Solanaceae family and Solanum genus phytochemicals studied are shown in Table 1 and their structures are shown in Figure 1. The alkaloids incanumine and solaradixine showed high binding energies to Mpro; the respective values were −9.8 and −9.4 kcal/mol. Two other alkaloids, khasianine and solasonine, also demonstrated high binding energies of −9.2 kcal/mol versus the antiviral drug lopinavir at −8.2 kcal/mol. Apart from solsodomine A with a binding energy of −5.1 kcal/mol, the other alkaloids also demonstrated high binding affinities of −7.5 kcal/mol or above. We did not observe any striking differences between the binding energy (ΔG) values of the several phytochemicals and control compounds (the control compounds were reported phytochemical inhibitors of Mpro, synthetic inhibitors of Mpro, and several antiviral compounds) evaluated against the three Mpro Protein Data Bank (PDB) crystalline structures, namely 6LU7, 6LZE, and 7BRO (Table 1), signifying that the AutoDock Vina results were correct. To be noted is that 6LU7 and 6LZE are crystalline structures with bound inhibitors, from which the respective inhibitors were removed prior to our molecular docking studies; 7BRO gives the crystalline structure of Mpro only without any bound inhibitors which is the apo enzyme.

Comparisons of predicted binding energies were also done with the expected hemiacetal, hemiketal, chalcone, and quinonoidal forms of the various anthocyanins/anthocyanidins evaluated in the present study. The predicted binding energies and the structures of the hemiacetal, hemiketal, chalcone, and quinonoidal forms of the anthocyanin/anthocyanidin compounds are shown in Table 2.

The anthocyanidins delphinidin and petunidin also showed high binding energies for Mpro; the binding energies were more enhanced with their glycosidic derivatives (anthocyanins). Delphinidin gave a predicted binding energy of −7.4 kcal/mol, whereas a number of glycosidic derivatives of delphinidin gave predicted binding energies from −8.2 to −8.6 kcal/mol, the highest predicted binding energy of −8.6 kcal/mol being obtained with delphinidin-3-rutinoside. A similar result was obtained with petunidin (predicted binding energy at −7.5 kcal/mol) and petunidin-3-(p-coumaroylrutinoside)-5-glucoside (predicted binding energy at −8.6 kcal/mol). This aspect of higher binding affinity to the 3C-like protease when a glycosidic linkage is present in the ligand has been noted previously by us [12] and others [39]. It has further been observed that the presence of rhamnose or rutinoside (glucose-rhamnose) leads to higher binding energies [39].

Interestingly, contrary to expectations, the predicted binding energies did not vary greatly between the various pH-dependent chemical forms of the anthocyanin/anthocyanidins, as shown in Table 2. For instance, the anthocyanidin delphinidin in its PubChem, chalcone, hemiketal, hemiacetal, and two quinonoidal forms gave predicted binding energies ΔG of −7.4, −6.9, −7.2, −7.7, −7.3, and −6.9, respectively (range of −6.9 to −7.4, difference between low and high values of predicted binding energies ΔG being only −0.5 kcal/mol). The maximum range of ΔG values was observed with petunidin-3-(p-coumaroyl-rutinoside)-5-glucoside from −7.4 (hemiketal form) to −9.1 kcal/mol (quinonoid 2 form).

It is also of interest that the anthocyanins and anthocyanidins are present in Solanum melongena (eggplant), the fruits of which are edible and widely consumed in many parts of the world, China and India being the top two producers (total of 50.19 million tons produced in 2014) [40]. Eggplants rank among the top ten vegetables with high antioxidant activity because of their high content of phenolic compounds [41]. Notably, antioxidant therapy has been proposed as a useful complementary therapy for COVID-19 [42]. Regarding Lipinski’s rule of five, alkaloids that were seen to maintain all the rules included capsimine, daturaolone, solanocapsine, solacasine, solacapine, episolacapine, and solsodomine A. These alkaloids had predicted binding energies to Mpro of −7.5, −8.1, −8.3, −8.1, −7.6, −7.9, and −5.1, respectively with the number of violations equal to zero. However, among these alkaloids, solsodomine A had a low predicted binding energy of only −5.1 kcal/mol, and so can possibly be discarded from further consideration as a drug molecule.

Delphinidin, delphinidin-3-glucoside, and petunidin were the only three non-alkaloid compounds where the number of violations to Lipinski’s rule did not exceed two, with one, two, and zero violations, respectively. The predicted binding energies of these three compounds were, respectively, −7.4, −8.4, and −7.5. As such, these compounds can make good drug candidates for COVID-19. The results are shown in Table 3 and agree well with previous studies [43].

Like Mpro of SARS, Mpro of SARS-CoV-2 is catalytically active as a dimer. Each monomeric unit contains three domains, namely, domain I consisting of amino acid residues 10–99, domain II (amino acid residues 100–182), and domain III (amino acid residues 198–303) [44]. Although domain III does not directly participate in interacting with the substrate, removal of domain III results in an inactive protease for domain III is involved in regulating dimerization of Mpro [45] and dimerization is necessary for the protease to be catalytically active [46]. A catalytic dyad is formed in the protease by Cys145 and His41. An irreversible inhibitor of Mpro of SARS-CoV-2, N3 was found to act by first attaching to the active site and then forming a covalent bond with Cys145 [47].

Besides His41 and Cys145, Met49 residue is necessary for substrate binding in SARS-CoV [48]. Additionally, Gly143, Ser144, His163, His164, Met165, Glu166, Leu167, Asp187, Arg188, Gln189, Thr190, Ala191, and Gln192 residues are also crucial for substrate binding in SARS-CoV Mpro [49,50], and these residues are conserved in SARS-CoV-2 Mpro [51].

**Figure 2:**
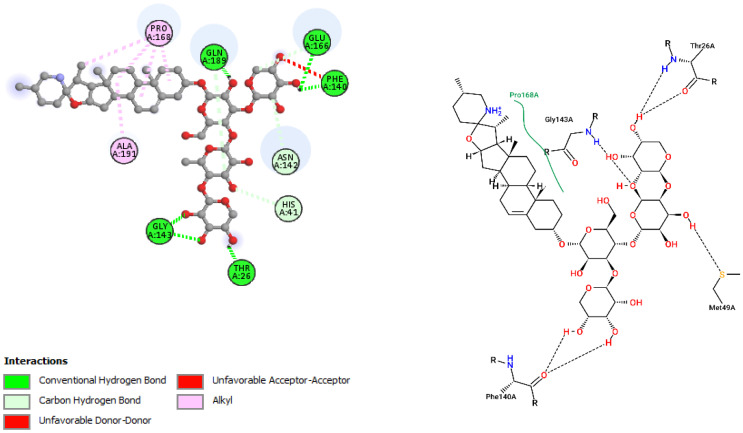
Interaction of incanumine with Mpro (without N3).

**Figure 3:**
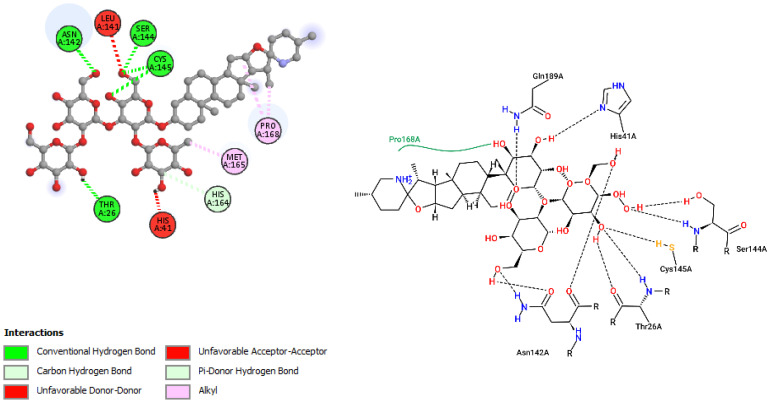
Interaction of solaradixine with Mpro (without N3).

**Figure 4:**
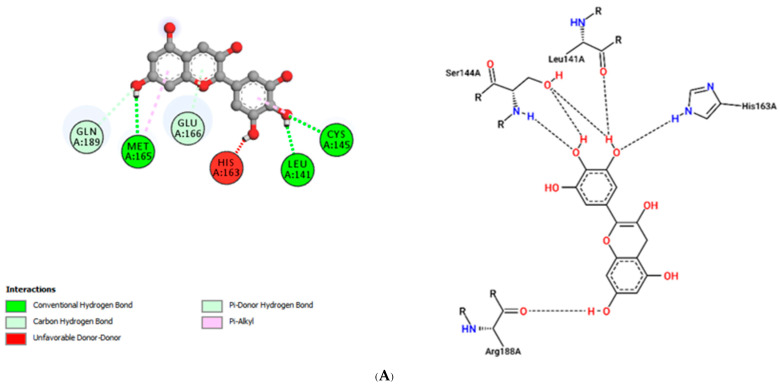

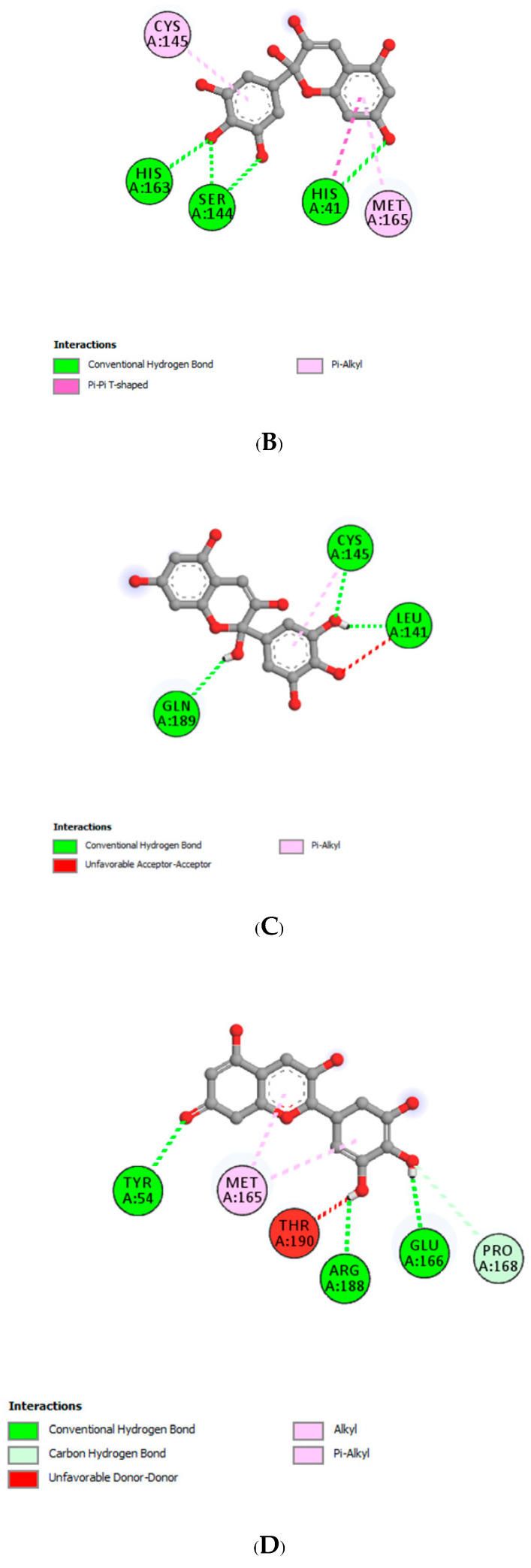

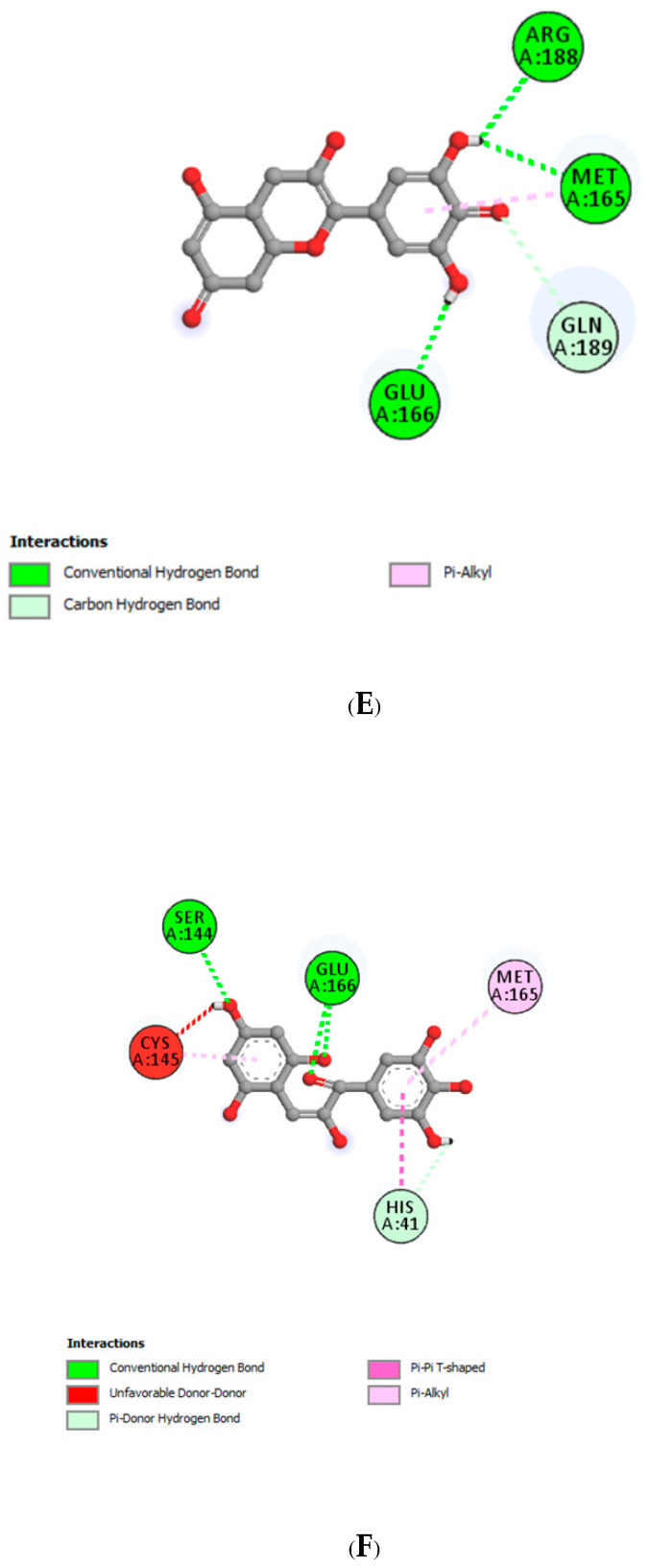
(**A**) Interaction of delphinidin (PubChem structure) with Mpro (without N3). (**B**) Interaction of delphinidin (hemiacetal form) with Mpro (without N3). (**C**) Interaction of delphinidin (hemiketal form) with Mpro (without N3). (**D**) Interaction of delphinidin (quinonoid 1 form) with Mpro (without N3). (**E**) Interaction of delphinidin (quinonoid 2 form) with Mpro (without N3). (**F**) Interaction of delphinidin (chalcone form) with Mpro (without N3).

**Figure 5:**
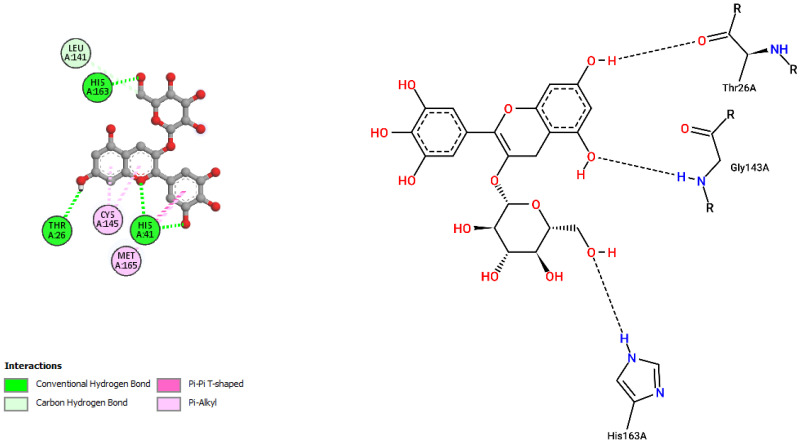
Interaction of delphinidin-3-glucoside with Mpro (without N3).

The interactions of incanumine, solaradixine, delphinidin, and delphinidin-3-glucoside with SARS-CoV-2 Mpro are shown in Figure 2, Figure 3, Figure 4 and Figure 5, since from Table 1, these four phytochemicals appeared to be of most interest. That of delphinidin is shown along with its various hemiacetal/hemiketal, quinonoidal, and chalcone forms (Figure 4).

Incanumine interacts with Thr26, **His41**, Phe140, Asn142, **Gly143**, **Glu166**, Pro168, **Gln189**, and **Ala191** (amino acids important for substrate binding and formation of catalytic dyads are given in bold), that is it interacts primarily with domain 2 of Mpro but also with the catalytic site (His41). Solaradixine interacts with Thr26, **His41**, Leu141, Asn142, **Ser144**, **Cys145**, **His164**, **Met165**, and Pro168, that is with the catalytic dyad amino acids as well as domain 2 amino acids. Interaction of delphinidin occurs with amino acid residues Leu141, **Cys145**, **His163**, **Met165**, **Glu166**, and **Gln189**, the amino acids being important for substrate binding or formation of catalytic dyads. Delphinidin-3-glucoside interacts with both the catalytic dyad amino acids as well as domain 2 amino acid residues involving Thr26, **His41**, Leu141, **Cys145**, **His163**, and **Met165**. It is to be noted that delphinidin-3-glucoside interacts with both His41 and Cys145 versus only Cys145 for delphinidin, which makes for a greater binding affinity of delphinidin-3-glucoside to Mpro. The non-bonded interactions of incanumine, solaradixine, delphinidin, delphinidin-3-glucoside, and several other phytochemicals of interest with SARS-CoV-2 Mpro amino acids are shown in Table 4. A comparative non-bonded interaction of delphinidin (PubChem derived) and its hemiacetal, hemiketal, chalcone, and two quinonoidal forms forming conventional hydrogen bond and carbon–hydrogen bond with Mpro (6LU7) amino acid residues is shown in Table 5.

Table 5 shows the Mpro (of PDB 6LU7) amino acids interacting through conventional hydrogen bonds and carbon–hydrogen bonds with the classical (PubChem) structure of delphinidin and its various pH-based chemical forms including the hemiacetal, hemiketal, chalcone, and two quinonoidal structures. It is evident that despite pH-based changes, the various pH-dependent forms of delphinidin interact with one or both amino acids of the catalytic dyad of Mpro as well as other amino acid residues important for substrate binding and catalytic function of Mpro. This interaction offers a possible explanation behind the quite similar predicted binding energies of different pH-dependent forms of the anthocyanins/anthocyanidins with Mpro as observed in the present study.

**Figure 6:**
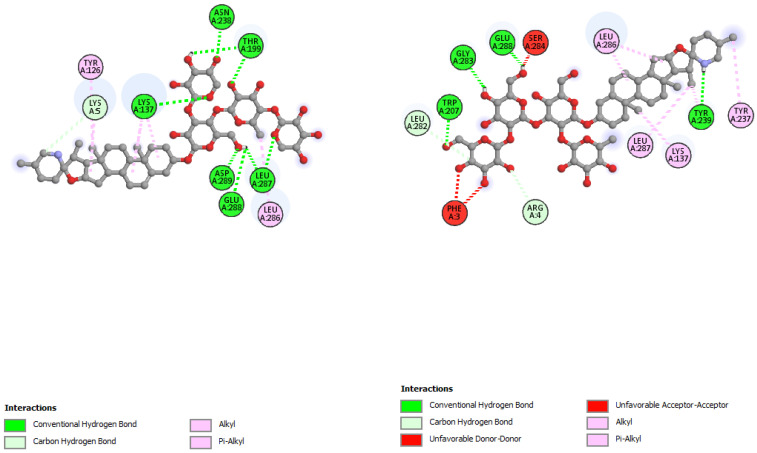
Interaction of incanumine (**left**) and solaradixine (**right**) with Mpro (with bound inhibitor N3).

**Figure 7:**
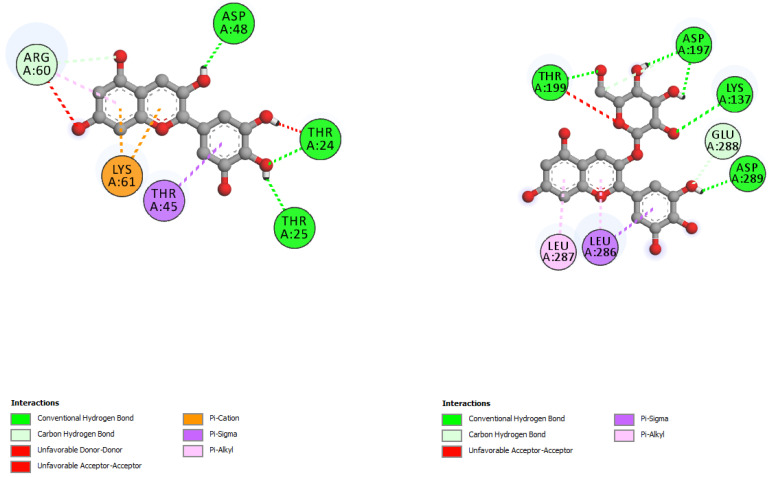
Interaction of delphinidin (**left**) and delphidin-3-glucoside (**right**) with Mpro (with bound inhibitor N3).

That the binding of incanumine, solaradixine, delphinidin, and delphinidin-3-glucoside was at the site of binding of the irreversible inhibitor N3 can also be seen in Figure 6 and Figure 7, where N3 binding to Mpro displaced the molecules from their original binding sites (compare Figure 6 and Figure 7 to Figure 2, Figure 3, Figure 4 and Figure 5).

The interacting residues of N3 with the protease amino acids include His41, Met49, Phe140, Leu141, Asn142, Gly143, His163, His164, Glu166, Leu167, Pro168, Gln189, Thr190, and Ala191 [52]. The interacting residues of incanumine, solaradixine, delphinidin, and del-phinidin-3-glucoside in the absence and presence of N3-bound Mpro are clearly delineated in Table 6 based on Figure 2, Figure 3, Figure 4, Figure 5, Figure 6 and Figure 7. It is obvious from Table 6 that apart from delphinidin, which compound’s binding shifts to domain 1 with N3-bound Mpro, the other three compounds’ bindings shift mainly to domain 3 with N3-bound Mpro. This displacement in binding was seen to lead to a decrease in binding energy for all four phytochemicals. Taken together, the results demonstrate two things, namely the phytochemicals can bind to different sites of Mpro and that their main binding site is to or close to where N3 binds to Mpro. It is therefore expected that the phytochemicals can be active inhibitors of Mpro.

**Figure 8:**
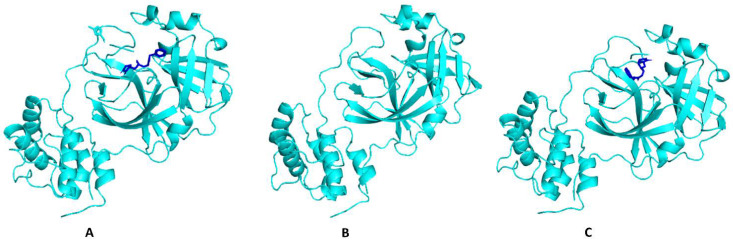
**(A**) Original 6LU7 (Mpro with bound N3), (**B**) Mpro (6LU7) without N3, and (**C**) N3 re-docked with apo-Mpro.

A final question remained as to how far our molecular docking was correct based on the fact that we took an inhibitor (N3) bound Mpro, removed the N3, and docked our phytochemicals and other compounds against the now N3-free Mpro? In Table 7 we show the amino acid residues with which N3 interacted in the original structure of Mpro (6LU7) and the amino acid residues with which N3 interacted in the re-docked Mpro. In Figure 8, we show the crystal structures of Mpro + N3, (Mpro from which N3 has been removed, and re-docked N3 with Mpro). There were no significant differences in the binding of N3 originally to Mpro and to re-docked Mpro. A notable feature was that in both structures, N3 interacted with one of the amino acids of the catalytic dyad of Mpro, namely HIS41. Other common interacting amino acid residues between the two structures of Mpro and N3 are depicted in red.

**Figure 9:**
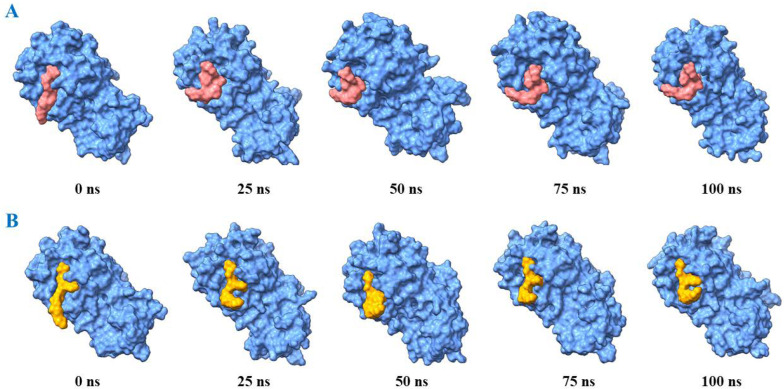
Binding poses of solaradixine and incanumine over the course of 100 ns simulation. The crystal structure of the main protease (PDB: 6LU7) is shown as a blue surface with solaradixine (orange) in (**A**) and incanumine (yellow) in (**B**) respectively.

A molecular dynamics (MD) simulation for complex structures (solaradixine-Mpro complex and incanumine-Mpro complex) and APO form (Mpro only) was performed for 100 ns. Figure 9 depicts that solaradixine and incanumine gave the poses in a stable position during the whole simulation run. Results obtained from MD simulation show that the RMSD (0.3–4.4 Å) of α-carbon atoms in APO protein (without compound) was higher than solaradixine and incanumine–protein complex structures. This suggests that APO protein was unstable in physiological conditions. In the case of the incanumine–protein complex, the RMSD (0.4–3.4 Å) was increased after 1.5 ns and decreased after 79 ns whereas the RMSD (0.4–3.2 Å) in the solaradixine–protein complex remained stable. Figure 9A indicates that incanumine exhibits more fluctuation than solaradixine during that time step. However, both complexes showed structural stability after 80 ns in physiological conditions. Moreover, the average RMSD for solaradixine was 2.1 Å and the average RMSD for incanumine was found to be 2.4 Å.

**Figure 10:**
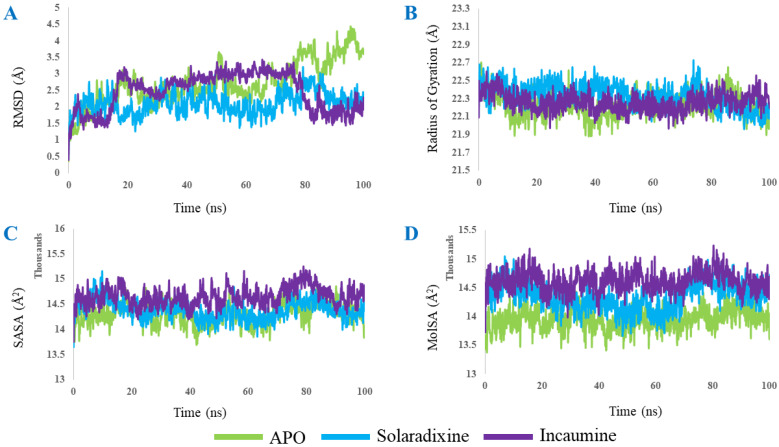
RMSD, radius of gyration, SASA, and MolSA values of Mpro without ligand (APO), solaradixine, and incanumine. (**A**) RMSD, or root-mean-square deviation, is a standard measure of structural distance between coordinates. (**B**) The radius of gyration (Rg) is defined as the distribution of atoms of a protein around its axis. (**C**) Solvent-accessible surface area (SASA) is the surface area of a biomolecule that is accessible to a solvent. (**D**) MolSA stands for molecular surface area.

The radius of gyration (Rg) was observed to describe the structural compactness of the phytochemical–protein complexes. The radius of gyration serves as an indicator of the compactness of protein structure [53,54]. It was found that a loose packing from the initial run to the 81 ns simulation run was obtained for solaradixine (Figure 10B) compared to the Rg of incanumine. However, this loose packing in the case that solaradixine becomes less after 81 ns which suggests that the solaradixine–protein complex structure is more compact and hence, more stable. The average radius of gyration of solaradixine and incanumine–protein complex structures was the same at 22.3 Å.

The solvent-accessible surface area (SASA) and molecular surface area (MolSA) were determined to figure out the solvent accessibility and the estimation of the protein surface of the drug–protein complexes. Figure 10C shows that the incanumine–protein complex exhibited higher solvent accessibility during the whole simulation run with an average value of 14660 Å2. In the case of the solaradixine–protein complex, SASA revealed comparatively lower solvent accessibility and the corresponding average value of SASA was 14387 Å2. Similarly, molecular surface area (MolSA) was also determined for the solaradixine and incanumine–protein complex structures. Figure 10D revealed that incanumine showed higher surface area (average 14620 Å2) during the whole run and solaradixine showed comparatively lower MolSA (average 14346 Å2). In the case of APO, SASA and MolSA covered the least surface area and the corresponding values were 14312 Å2 and 13950 Å2, respectively compared to those of solaradixine and incanumine–protein complex structures.

Except for solsodomine A, the rest of the phytochemicals of various Solanaceae family plants evaluated in the present study demonstrated low binding energies that are high binding affinities to Mpro of SARS-CoV-2. As shown in Table 3, of the six phytochemicals shown, all six compounds interacted with either His41 or Cys145 or both amino acid residues of the catalytic dyad, suggesting that they would make good inhibitors of Mpro. Since Mpro is a vital protease for replication of SARS-CoV-2, any inhibition of Mpro will lead to the stoppage of viral replication. Delphinidin and its derivatives demonstrated high binding affinities for Mpro. Delphinidin has previously been reported to demonstrate virucidal activity against West Nile, dengue, and Zika virus [55].

The two phytochemicals having the highest predicted binding energy, namely solaradixine and incanumine were chosen for the molecular dynamics (MD) simulation. It was found that the average RMSD of solaradixine (2.1 Å) was slightly lower than that of incanumine (2.4 Å). This indicated that the solaradixine–protein complex was more stable during the whole simulation run. The radius of gyration (Rg) results depicted that solaradixine exhibited more compactness after the 81 ns run compared to incanumine, which indicated that solaradixine bound closer to Mpro and was more stable. SASA and MolSA revealed that the covered surface area was less for solaradixine than incanumine. This data also gave further insights into the tighter binding of solaradixine to the protein. So, the results of RMSD, Rg, SASA, and MolSA performed by MD simulation support the docking results which convey that the selected drugs actively interacted with the main protease, Mpro. Since the present work dealt only with in silico studies, various types of validations were made of the results obtained from initial molecular docking experiments. These validations included (I) performing molecular docking experiments with other crystalline structures of Mpro including the apo enzyme; (II) using known synthetic and phytochemical inhibitors (previously published) of Mpro and checking previous molecular docking results with our re-docked results; (III) taking an inhibitor (N3) bound Mpro-taking the inhibitor off- and redocking N3 to the apo enzyme to see whether it docked to the previous site on Mpro; (IV) last but not least, molecular dynamics studies. All results are in agreement with each other, validating the present study.

## 4. Conclusions

**Figure 11:**
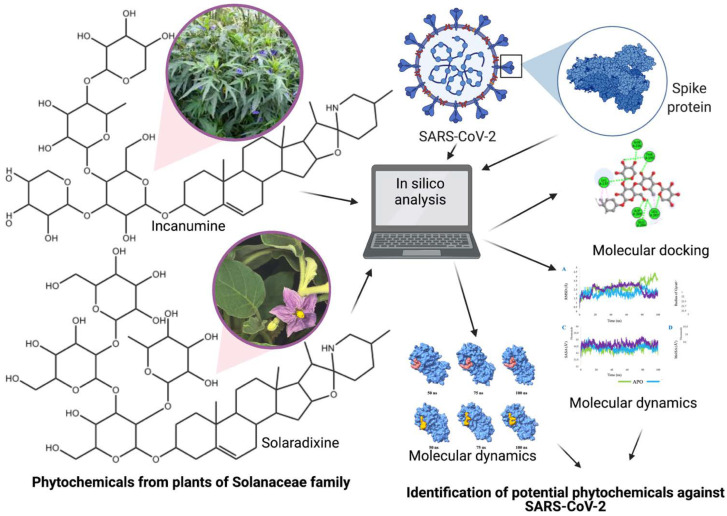
Identification of potential compounds against SARS-CoV-2 using in silico studies.

The results presented in this study strongly suggest that several phytochemicals from the Solanaceae family can be drug candidates or act as lead compounds against SARS-CoV-2 through binding to the virus’s main protease, Mpro (Figure 11); also, to be noted is that the bindings of the phytochemicals to Mpro appear to be quite specific as demonstrated by equivalent binding energies between randomly selected phytochemicals and two different X-ray structures of Mpro. If the in silico results can be confirmed through further antiviral in vitro and in vivo studies, this can be beneficial from several viewpoints. Drugs are usually preferred by people when compared to vaccines because of an inherent fear of injections and needles, and in this case, any drugs derived from edible Solanaceae family fruits or vegetables will be welcomed because of the edible nature of the plant parts. Second, it is quite possible that even if the drugs are synthesized chemically, they will be more affordable and less cumbersome to store. Anthocyanins and anthocyanidins can provide additional benefits to COVID-19 patients through their diverse pharmacological activities, one of the most important being reducing oxidative stress.

## Figures and Tables

**Table 1:** Predicted binding energy in molecular docking studies of some Solanaceae family phytochemicals to Mpro (without attached inhibitor) of SARS-CoV-2. Additionally presented are the binding energies of several drugs used against COVID-19 and flavonoids reported to bind to Mpro in in silico studies. Note that three reported crystalline structures of Mpro were used, namely 6LU7, 6LZE, and 7BRO for comparison of results of binding energies.

Phytochemical	Source	Binding Energy (ΔG = kcal/mol)		
		6LU7	6LZE	7BRO
Incanumine	*Solanum incanum* L.	−9.8	−9.3	
Isocapsicastrine	*Solanum capsicastrum* Link ex Schauer.	−8.4		
Khasianine	*Solanum xanthocarpum* Schrad. and Wendl.	−9.2	−8.8	
Solaradixine	*Solanum laciniatum* Aiton.	−9.4	−8.3	
Solasonine	*Solanum asperum* Rich.	−9.2	−9.3	
Capsimine	*Solanum capsicastrum* Link ex Schauer.	−7.5		
Daturaolone	*Solanum arundo* Mattei	−8.1		−8.3
Solanocapsine	*Solanum capsicastrum* Link ex Schauer.	−8.3		
Solacasine	*Solanum capsicastrum* Link ex Schauer.	−8.1		
Solacapine	*Solanum capsicastrum* Link ex Schauer.	−7.6	−7.7	
Episolacapine	*Solanum capsicastrum* Link ex Schauer.	−7.9		
Solsodomine A	*Solanum sodomeum* L.	−5.1	−5.1	
Delphinidin	*Solanum melongena* L.	−7.4		−7.3
Nasunin (delphinidin-3-*p*-coumaroylrutinoside-5-glucoside)	*Solanum melongena* L.	−8.5		
Delphinidin-3-rutinoside (Tulipanin)	*Solanum melongena* L.	−8.6		
Delphinidin-3-rutinoside-5-glucoside	*Solanum melongena* L.	−8.5		
Delphinidin-3-glucoside (Myrtillin/Mirtillin)	*Solanum melongena* L.	−8.4		
Delphinidin-3-(caffeoyl-rutinoside)-5-glucoside	*Solanum melongena* L.	−8.2		
Petunidin-3-(p-coumaroylrutinoside)-5-glucoside	*Solanum melongena* L.	−8.6		
Petunidin	*Solanum melongena* L.	−7.5	−7.3	
Lopinavir	Antiviral control drug	−8.2		
Baricitinib	Antiviral control drug	−6.6	−7.2	
Capsaicin	Alkaloid in *Capsicum annuum* L.	−5.9	−5.8	
Ivermectin	Anti-parasitic control drug	−9.1	−8.8	
Molnupiravir	Antiviral control drug	−6.6	−6.4	
Quercetin	Flavonoid found in grapes	−6.6	−7.3	
Luteolin	Reported Mpro inhibitor	−7.5	−7.5	
Remdesivir	Antiviral control drug	−7.4	−7.8	
Nirmatrelvir	Antiviral control drug	−7.5		−7.0
Bedaquiline	Mpro inhibitor	−6.9		−6.8
Boceprevir	Mpro inhibitor	−6.4		−6.9
Efonidipine	Mpro inhibitor	−7.5		−7.3
Lercanidipine	Mpro inhibitor	−7.8		−7.3
Manidipine	Mpro inhibitor	−7.2		−6.5

**Table 2:** Structures and predicted binding energies of different pH-based forms of anthocyanins and anthocyanidins and Mpro (6LU7 without bound N3 inhibitor) of SARS-CoV-2.

Name of the Chemicals	Chemical Structure	ΔG = kcal/mol
Delphinidin (PubChem)	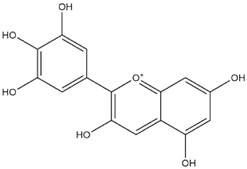	−7.4
Delphinidin-E-Chalcone	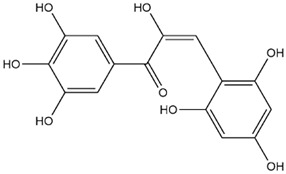	−6.9
Delphinidin Hemiketal	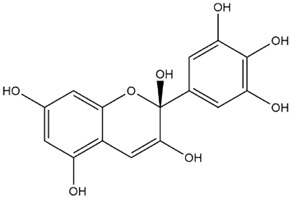	−7.2
Delphinidin Hemiacetal	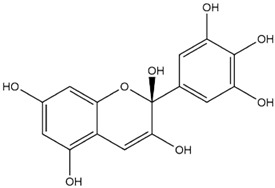	−7.7
Delphinidin Quinonoid-1	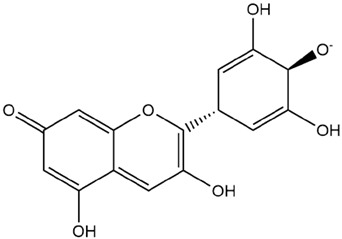	−7.3
Delphinidin Quinonoid-2	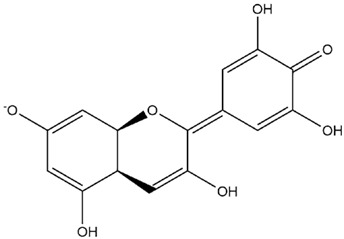	−6.9
Delphinidin-3-glucoside (PubChem)	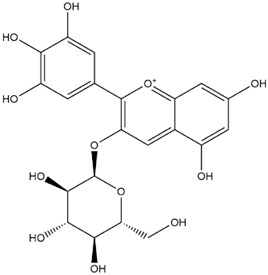	−8.4
Mirtillin-E-Chalcone (Delphinidin-3-glucoside chalcone form)	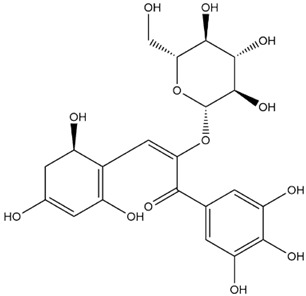	−7.9
Mirtillin Quinonoid-1	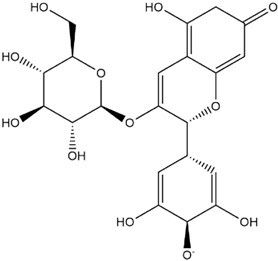	−8.3
Mirtillin Quinonoid-2	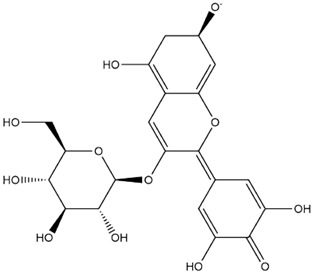	−7.6
Nasunin (PubChem)	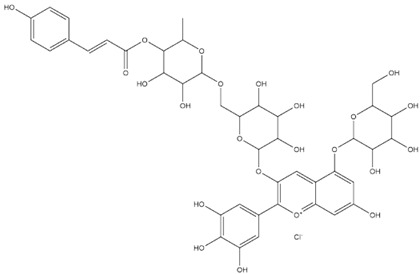	−8.5
Nasunin-E-Chalcone	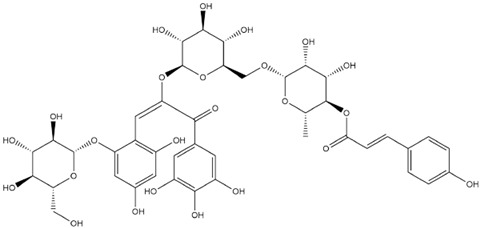	−8.5
Nasunin Hemiketal	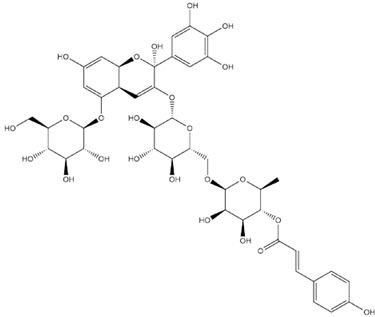	−7.3
Nasunin Quinonoid-1	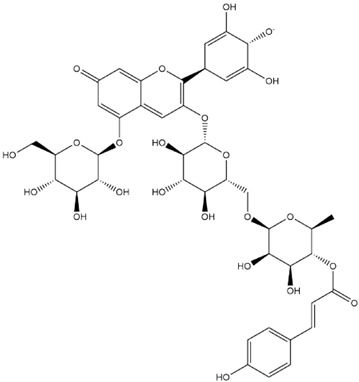	−8.3
Nasunin Quinonoid-2	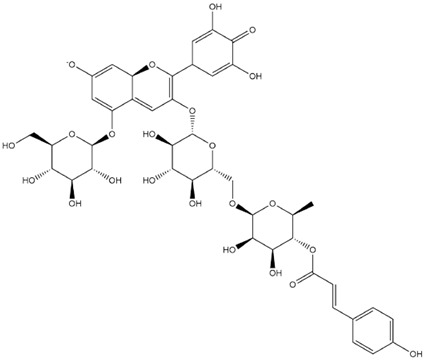	−8.5
Petunidin (PubChem)	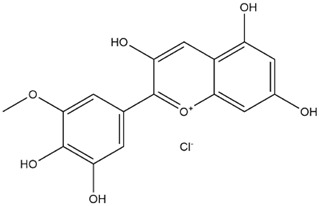	−7.5
Petunidin-E-Chalcone	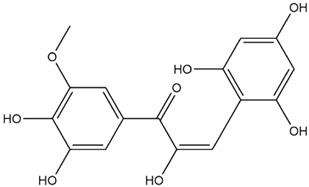	−7.1
Petunidin Hemiketal	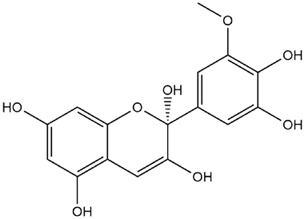	−7.6
Petunidin Quinonoid-1	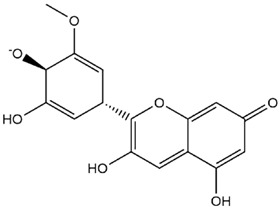	−7.3
Petunidin Quinonoid-2	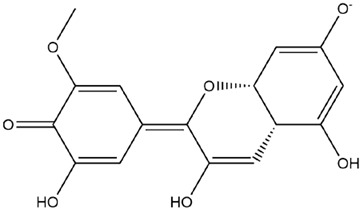	−6.5
Delphinidin-3-rutinoside (PubChem)	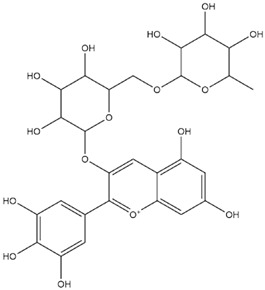	−8.6
Tulipanin-E-Chalcone (Delphinidin-3-rutinoside chalcone form)	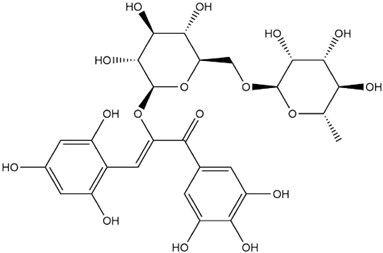	−7.9
Tulipanin Hemiketal	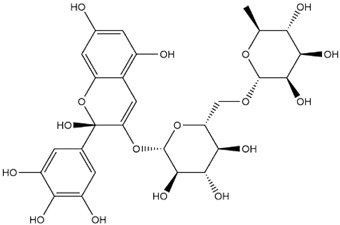	−8.1
Tulipanin Quinonoid-1	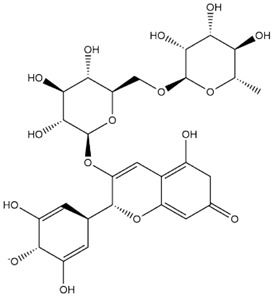	−9.4
Tulipanin Quinonoid-2	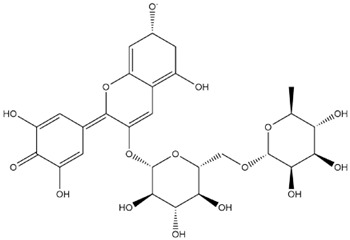	−8.2
Delphinidin-3-(Caffeoyl-Rutinoside)-5-Glucoside (PubChem)	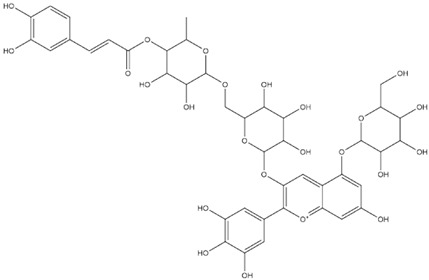	−8.2
Delphinidin-3-Caffeoyl-Rutinoside-5-Glucoside-E-Chalcone	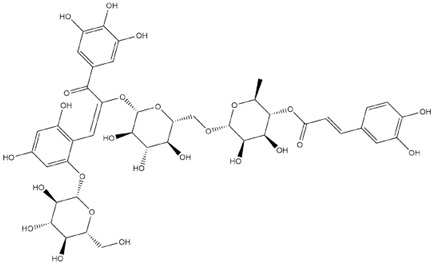	−8.2
Delphinidin-3-Caffeoyl-Rutinoside-5-Glucoside Hemiketal	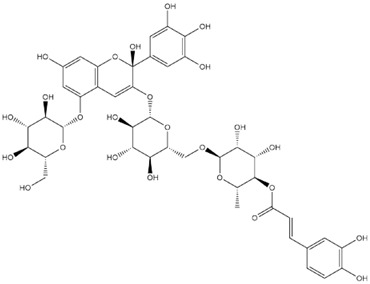	−8.5
Delphinidin-3-Caffeoyl-Rutinoside-5-Glucoside Quinonoid-1	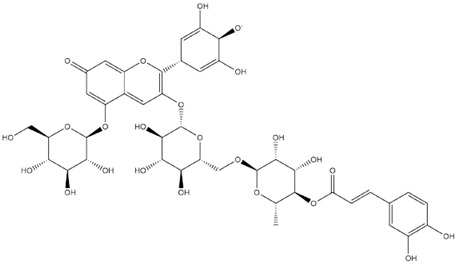	−8.8
Delphinidin-3-Caffeoyl-Rutinoside-5-Glucoside Quinonoid-2	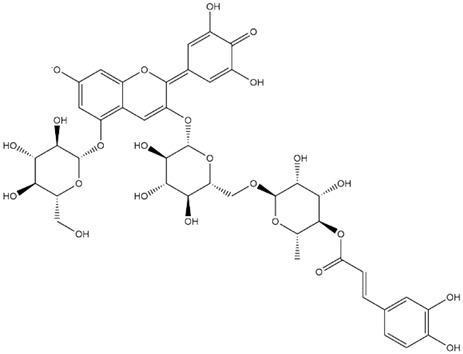	−8.0
Delphinidin-3-Rutinoside-5-Glucoside (PubChem)	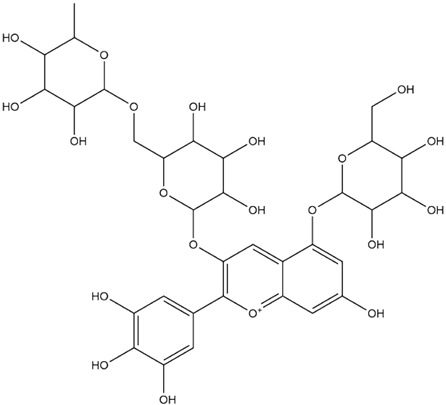	−8.5
Delphinidin-3-Rutinoside-5-Glucoside-E-Chalcone	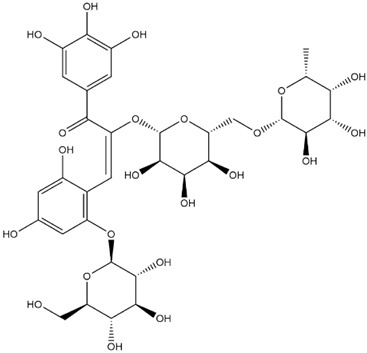	−8.2
Delphinidin-3-Rutinoside-5-Glucoside Hemiketal	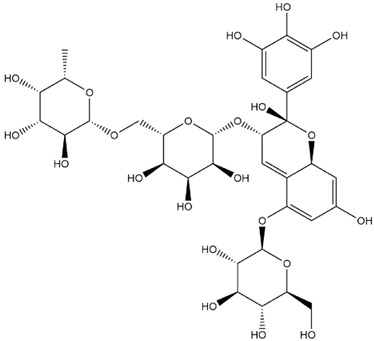	−8.4
Delphinidin-3-Rutinoside-5-Glucoside Quinonoid-1	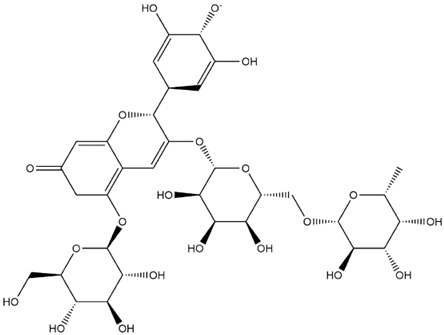	−7.8
Delphinidin-3-Rutinoside-5-Glucoside Quinonoid-2	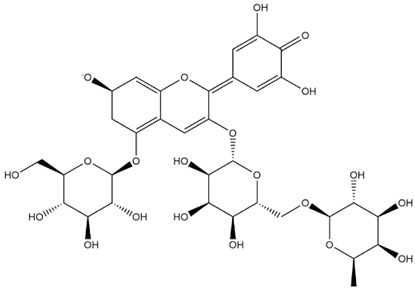	−8.6
Petunidin-3-(*p*-Coumaroyl-Rutinoside)-5-Glucoside	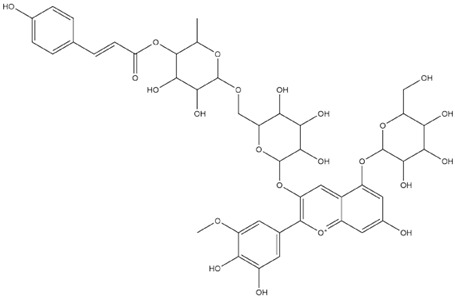	−8.6
Petunidin-3-(p-Coumaroyl-Rutinoside)-5-Glucoside Chalcone	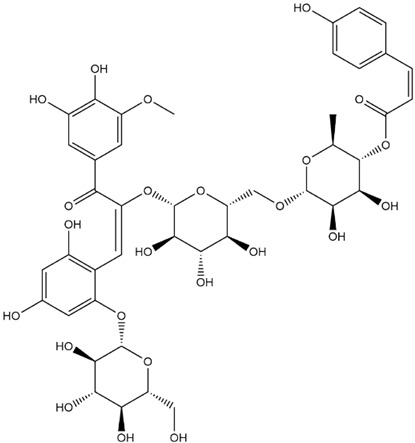	−8.3
Petunidin-3-(p-Coumaroyl-Rutinoside)-5-Glucoside Hemiketal	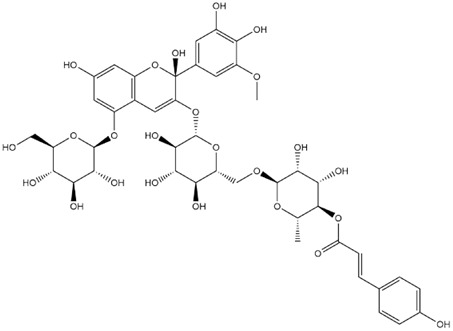	−7.4
Petunidin-3-(p-Coumaroyl-Rutinoside)-5-Glucoside Quinonoid-1	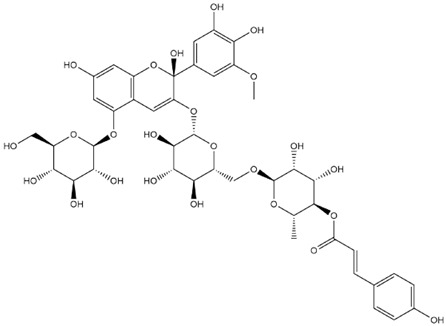	−8.7
Petunidin-3-(p-Coumaroyl-Rutinoside)-5-Glucoside Quinonoid-2	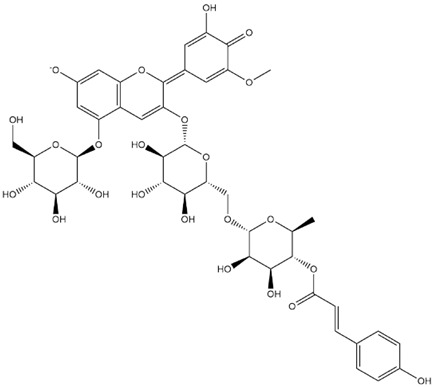	−9.1

**Table 3:** Physicochemical properties of some Solanaceae family phytochemicals.

Phytochemical (Binding Energy in kcal/mol)	Molecular Weight	Number of H-Bond Acceptors	Number of H-Bond Donors	Log P	Molar Refractivity	Number of Violations of Rule of Five
Incanumine (−9.8)	986.15	20	11	4.65	243.66	4
Isocapsicastrine (−8.4)	577.79	8	6	4.42	161.76	3
Khasianine (−9.2)	721.92	12	7	4.74	190.83	5
Solaradixine (−9.4)	1046.20	22	13	4.09	255.60	4
Solasonine (−9.2)	884.06	17	10	4.03	223.21	4
Capsimine (−7.5)	415.65	3	3	4.22	129.38	0
Daturaolone (−8.1)	440.70	2	1	4.37	135.08	0
Solanocapsine (−8.3)	430.67	4	3	4.14	130.41	0
Solacasine (−8.1)	442.68	4	1	4.53	135.43	0
Solacapine (−7.6)	432.68	4	4	3.85	132.56	0
Episolacapine (7.9)	442.68	4	1	4.53	135.43	0
Solsodomine A (−5.1)	204.23	2	2	0.91	57.03	0
Delphinidin (−7.4)	303.24	7	6	−3.10	78.20	1
Nasunin (delphinidin-3-p-coumaroylrutinoside-5-glucoside (−8.5)	955.26	23	14	−5.04	220.89	4
Delphinidin-3-rutinoside (−8.6)	611.53	16	11	−2.34	141.54	3
Delphinidin-3-rutinoside-5-glucoside (−8.5)	773.67	21	14	−1.98	173.66	3
Delphinidin-3-glucoside (−8.4)	500.84	12	9	−5.34	116.17	2
Delphinidin-3-(caffeoyl-rutinoside)-5- Glucoside (−8.2)	935.81	24	15	−0.74	217.06	4
Petunidin-3-(p-coumaroylrutinoside)-5- Glucoside (−8.6)	933.84	23	13	0.49	219.50	4
Petunidin (−7.5)	317.27	7	5	−1.72	85.66	0

**Table 4:** Non-bonded interactions of some Solanaceae family phytochemicals with the 3C-like protease of SARS-CoV-2. (CH = conventional hydrogen bond; C = carbon–hydrogen bond).

Incanumine			
Residues	Distance	Category	Type
GLY143	2.68	Hydrogen Bond	CH
GLY143	2.14	Hydrogen Bond	CH
GLN189	1.87	Hydrogen Bond	CH
PHE140	2.09	Hydrogen Bond	CH
GLU166	2.51	Hydrogen Bond	CH
THR26	2.45	Hydrogen Bond	CH
HIS41	3.75	Hydrogen Bond	C
GLU166	3.25	Hydrogen Bond	C
ASN142	3.23	Hydrogen Bond	C
GLN189	3.40	Hydrogen Bond	C
PRO168	4.84	Hydrophobic	Alkyl
PRO168	4.67	Hydrophobic	Alkyl
PRO168	4.65	Hydrophobic	Alkyl
ALA191	4.80	Hydrophobic	Alkyl
PRO168	4.33	Hydrophobic	Alkyl
**Solaradixine**			
ASN142	2.26	Hydrogen Bond	CH
SER144	2.38	Hydrogen Bond	CH
CYS145	2.67	Hydrogen Bond	CH
CYS145	2.76	Hydrogen Bond	CH
THR26	1.97	Hydrogen Bond	CH
HIS164	3.32	Hydrogen Bond	C
HIS41	2.33	Hydrogen Bond	Pi-Donor Hydrogen Bond
PRO168	4.52	Hydrophobic	Alkyl
PRO168	4.31	Hydrophobic	Alkyl
MET165	4.16	Hydrophobic	Alkyl
**Daturaolone**			
ASN142	3.63	Hydrogen Bond	C
PRO168	4.79	Hydrophobic	Alkyl
CYS145	4.64	Hydrophobic	Alkyl
CYS145	4.09	Hydrophobic	Alkyl
PRO168	4.47	Hydrophobic	Alkyl
**Delphinidin**			
LEU141	1.79	Hydrogen Bond	CH
MET165	2.76	Hydrogen Bond	CH
GLN189	3.27	Hydrogen Bond	CH
GLU166	3.07	Hydrogen Bond	Pi-Donor Hydrogen Bond
MET165	5.31	Hydrophobic	Pi-Alkyl
CYS145	4.86	Hydrophobic	Pi-Alkyl
CYS145	2.63	Hydrogen Bond	CH
**Delphinidin** **-** **3** **-** **glucoside**			
HIS41	3.30	Hydrogen Bond	CH
HIS41	2.96	Hydrogen Bond	CH
HIS163	1.89	Hydrogen Bond	CH
THR26	2.37	Hydrogen Bond	CH
LEU141	3.43	Hydrogen Bond	C
HIS41	4.78	Hydrophobic	Pi-Pi T-shaped
CYS145	4.88	Hydrophobic	Pi-Alkyl
CYS145	4.88	Hydrophobic	Pi-Alkyl
MET165	5.10	Hydrophobic	Pi-Alkyl
**Delphinidin** **-** **3** **-** **rutinoside**			
CYS145	2.95	Hydrogen Bond	CH
THR26	2.73	Hydrogen Bond	CH
THR26	2.75	Hydrogen Bond	CH
GLU166	2.58	Hydrogen Bond	CH
CYS145	4.97	Other	Pi-Sulfur
MET49	5.09	Hydrophobic	Pi-Alkyl
MET49	4.66	Hydrophobic	Pi-Alkyl

**Table 5:** Non-bonded interactions of delphinidin and several of its pH-based structural forms with the 3C-like protease of SARS-CoV-2 (PDB 6LU7) amino acid residues.

Interacting Amino Acid Residues of Mpro with					
Delphinidin (PubChem)	Delphinidin Hemiacetal	Delphinidin Hemiketal	Delphinidin Quinonoid 1	Delphinidin Quinonoid 2	Delphinidin Chalcone
LEU141	HIS41	CYS145	TYR54	GLU166	SER144
MET165	SER144	LEU141	ARG188	MET165	GLU166
GLN189	HIS163	GLN189	GLU166	ARG188	HIS41
GLU166	MET165		PRO168	GLN189	MET165
MET165	CYS145		MET165	MET165	CYS145
CYS145					

**Table 6:** Binding of incanumine, solaradixine, delphinidin, and delphinidin-3-glucoside with Mpro and N3-bound Mpro.

Phytochemicals	Interaction with Mpro	Binding Energy (ΔG = kcal/mol)	Interaction with N3-Mpro	Binding Energy (ΔG = kcal/mol)
N3 (irreversible inhibitor)	His41, Met49, Phe140, Leu141, Asn142, Gly143, His163, His164, Glu166, Leu167, Pro168, Gln189, Thr190, Ala191		Not applicable	
Incanumine	Thr26, His41, Phe140, Asn142, Gly143, Glu166, Pro168, Gln189, Ala191	−9.8	Lys5, Tyr126, Lys137, Thr199, Asn238, Leu286, Leu287, Glu288, Asp289	−8.9
Solaradixine	Thr26, His 41, Leu141, Asn142, Ser144, Cys145, His164, Met165, Pro168	−9.4	Phe3, Arg4, Lys137, Trp207, Tyr237, Tyr239, Leu282, Gly283, Ser284, Leu286, Glu288	−8.1
Delphinidin	Leu141, Cys145, His163, Met165, Glu166, Gln189	−7.4	Thr24, Thr25, Thr45, Asp48, Arg60, Lys61	−6.9
Delphinidin−3-glucoside	Thr26, His41, Leu141, Cys145, His163, Met165	−8.4	Lys137, Asp197, Thr199, Leu286, Leu287, Glu288, Asp289	−7.2

**Table 7:** Non-bonded interactions of N3 with Mpro in the pdb structure (PDB: 6LU7) in both original and re-docked pose.

Residues	Distance	Bond Category	Type
**Non-bonded interactions of N3 with Mpro in the original pose.**			
GLY143	2.79	Hydrogen Bond	Conventional Hydrogen Bond
GLU166	2.97	Hydrogen Bond	Conventional Hydrogen Bond
THR190	2.84	Hydrogen Bond	Conventional Hydrogen Bond
GLU166	2.83	Hydrogen Bond	Conventional Hydrogen Bond
GLN189	2.93	Hydrogen Bond	Conventional Hydrogen Bond
PHE140	3.13	Hydrogen Bond	Conventional Hydrogen Bond
GLU166	3.38	Hydrogen Bond	Conventional Hydrogen Bond
HIS164	3.07	Hydrogen Bond	Conventional Hydrogen Bond
HIS172	3.32	Hydrogen Bond	Carbon–Hydrogen Bond
MET49	4.66	Hydrophobic	Alkyl
MET165	4.57	Hydrophobic	Alkyl
LEU167	5.46	Hydrophobic	Alkyl
HIS41	4.31	Hydrophobic	Pi-Alkyl
PRO168	4.84	Hydrophobic	Pi-Alkyl
ALA191	4.53	Hydrophobic	Pi-Alkyl
**Non-bonded interactions of N3 with Mpro in the re-docked pose.**			
HIS163	2.01	Hydrogen Bond	Conventional Hydrogen Bond
GLU166	2.32	Hydrogen Bond	Conventional Hydrogen Bond
GLN189	2.55	Hydrogen Bond	Conventional Hydrogen Bond
GLN189	2.27	Hydrogen Bond	Conventional Hydrogen Bond
GLU166	3.73	Hydrogen Bond	Carbon–Hydrogen Bond
GLU166	4.33	Electrostatic	Pi-Anion
HIS41	3.92	Hydrophobic	Pi-Sigma
MET49	3.89	Hydrophobic	Alkyl

## Data Availability

Not applicable.
